# Human vs. LLM Creativity: A Comparative Analysis of Task-Dependent Asymmetry and Linguistic Mechanisms

**DOI:** 10.3390/jintelligence14020027

**Published:** 2026-02-05

**Authors:** Liping Yang, Tao Xin, Yunye Yu, Yiying Wu

**Affiliations:** 1 Higher Education Supervision and Evaluation Institute, Beijing Institute of Educational Supervision and Evaluation, Beijing 100034, China; lipingyang@163.com; 2 Collaborative Innovation Center of Assessment for Basic Education Quality, Beijing Normal University, Beijing 100875, China; 3 Center for General Education, Westlake University, Hangzhou 310030, China; yuyunye@westlake.edu.cn; 4 School of Architecture, Design and Planning, The University of Sydney, Sydney 2016, Australia; yiying.wu@sydney.edu.au

**Keywords:** creativity assessment, large language models (LLMs), human–AI collaboration, linguistic mechanisms, task dependence

## Abstract

This study investigates the distinct mechanisms of human versus Large Language Model (LLM) creativity. Employing a two-stage experimental design, we systematically compared Human-Only, LLM-Only, and LLM-Assisted performance across propositional and creative writing tasks. Results revealed a critical asymmetry contingent upon the research context: human authors exhibited higher originality in high-demand creative tasks, whereas LLMs governed execution quality, maintaining superior effectiveness across different tasks and cohorts. This pattern is characterized by four exploratory writing creativity profiles: Ideal, Safe, Moderate, and Plain. The distribution of human and LLM writings across these profiles was strikingly different. Hierarchical Moderated Regression analysis uncovered divergent linguistic pathways: human originality is predicted by markers of subjective cognitive investment, while LLM effectiveness is mechanistically driven by optimized structural coherence. Furthermore, the study identified a “Collaboration Trap” during collaboration with a suboptimal LLM. This partnership failed to improve human performance relative to LLM-Only benchmarks and induced cognitive anchoring, leading humans to mimic AI complexity without quality gains. These insights offer critical implications for preserving human agency and avoiding homogenization in future human–AI collaborative writing pedagogies.

## 1. Introduction

For a long time, creativity has been regarded as a unique cognitive fortress of human beings, characterized as the crystallization of emotion, experience, and higher-order thinking (Boden, 2004; Runco & Jaeger, 2012). However, the emergence of Large Language Models (LLMs)—neural networks trained on internet-scale corpora—has compelled the academic community to re-examine the boundaries of creativity (Brown et al., 2020; Anthropic, 2024). LLMs can now produce not only grammatically fluent and logically coherent texts but also seemingly “creative” outputs in traditional creative domains, such as story writing and poetry generation. This technological breakthrough directly challenges the traditional notion that “creativity is uniquely human”.

This emerging paradigm has rendered the initial binary question “Can AI be creative?” increasingly obsolete, replacing it with a more critical and nuanced inquiry: “How does artificial creativity fundamentally differ from human creativity?” (Boden, 2004; Jordanous, 2012; Colton & Wiggins, 2012). This question is central because creativity itself is not a monolithic construct (Kaufman & Beghetto, 2009). Creativity has long been conceptualized through the “standard definition”, which includes the bipartite criteria of novelty and usefulness (Runco & Jaeger, 2012; Amabile, 1983; Stein, 1953). This dual-criterion definition has achieved broad consensus across psychology, education, and cognitive science (Diedrich et al., 2015). This essential requirement involves moving beyond simple divergent thinking tasks toward sophisticated domain-specific product evaluations. Originality refers to the generation of ideas that are unique, statistically infrequent, and surprising (Guilford, 1950; Simonton, 2012; Boden, 2004). Effectiveness denotes the requirement that these ideas must be appropriate, useful, and valuable or exhibit high coherence and fluency within a specific domain (such as writing), while adhering to task constraints (Runco & Jaeger, 2012; Cropley, 2006). However, this product-oriented consensus has recently come under significant theoretical scrutiny. Relying solely on external judgments of “usefulness” may fail to capture the internal cognitive experience of the creator. To address this, contemporary perspectives suggest shifting from a purely functional “usefulness” toward a broader sense of “satisfying” the creator’s intent, thereby encompassing both the generative act and the resulting product (Abraham, 2025).

For writing, evaluating human and AI-generated texts against the originality-effectiveness framework avoids the limitations of focusing solely on a single creativity score (Baer, 2012). This dual-dimensional framework is pivotal in distinguishing “genius creations” from “madman’s nonsense” (Simonton, 1999; Sternberg, 1999), while also serving as the benchmark for evaluating the integrative nature of writing creativity. A combination of originality without effectiveness is meaningless randomness, while effectiveness without originality constitutes routine competence (Cropley & Cropley, 2008; Runco & Charles, 1993). 

Following this introduction, Section 2 provides a systematic review of current advancements in LLM text generation and the emerging conflicts in human–AI comparative creativity, and then details the specific objectives and investigative strategies of this study based on the identified research gaps.

## 2. Literature Review

### 2.1. Creativity Performance of LLM Text Generation

The proliferation of LLMs, particularly those built on the Transformer architecture, has inaugurated a new era for the study of computational creativity.

#### 2.1.1. The Outbreak of Text Generation and Creativity

Since the release of GPT-3 and its subsequent models, LLMs have achieved remarkable advancements. LLMs have substantially lowered the barrier to content production, as they are capable of generating grammatically flawless and logically consistent text within extremely short timeframes. The mechanistic roots of this phenomenon lie in LLMs’ primary training objectives: perplexity minimization and Reinforcement Learning from Human Feedback (RLHF) (Ouyang et al., 2022). As models are trained to predict the “most probable” next token, their output inherently tends toward conformity with mainstream conventions, exhibiting rigorous logic and high comprehensibility (Bender et al., 2021). This lays the foundation for LLMs to possess writing creativity.

LLMs demonstrate highly efficient Divergent Thinking (DT) due to their powerful retrieval and combination capabilities (Vinchon et al., 2024). They can generate a large volume of diverse ideas in short periods and often match or exceed the quantity and diversity of top-performing human groups in classical DT tests, such as the Alternative Uses Task (AUT) (Koivisto & Grassini, 2023). This capacity stems from their ability to explore and recombine the vast semantic space embedded in their training data at minimal computational cost, substantially accelerating the rate of idea production (Haase & Hanel, 2023).

Crucially, AI-generated texts consistently exhibit fewer grammatical errors and possess a more refined structure than human-authored texts, both in scientific writing and in everyday conversation, directly elevating their effectiveness scores (Liang et al., 2023).

#### 2.1.2. Hidden Constraints and Latent Limitations

Large Language Models also have obvious limitations in writing creativity. AI tends to utilize high-frequency words and standard sentence structures, often eschewing low-frequency vocabulary or complex metaphors (Chang & Bergen, 2023), which, while highly expressive, might potentially lead to comprehension difficulties. LLMs exhibit “feature universality”, meaning that different models are highly aligned in semantic space. Consequently, texts generated through probabilistic computation often exemplify “combinatorial creativity” rather than “transformative creativity” (Boden, 2004; Wiggins, 2006). Furthermore, a significant gap remains in emotional depth and embodied cognition. Although AI can generate vocabulary expressing sadness, it fundamentally lacks the subtle subtext or complex socio-emotional interactions necessary to elicit deep resonance (Pavlik, 2023).

Additionally, regarding “hallucinations”, while some argue these may serve as a source of artistic inspiration, others demonstrate that in rigorous tasks, hallucinations lack the “plausible novelty” required for genuine discovery (Ji et al., 2023). This reiterates the constraining role of the “effectiveness” dimension in the standard definition: “originality” devoid of real-world logical constraints does not constitute genuine creativity.

### 2.2. Which Is Superior in Writing Creativity: Human or LLM?

The evaluation of creative outputs requires a dual-dimensional framework of originality (novelty, conceptual depth) and effectiveness (formality, coherence), a standard consensus in psychometric and language testing research. The reliability of scoring was primarily assessed using the Intraclass Correlation Coefficient (ICC), computed with a two-way random-effects model for absolute agreement among k raters (Shrout & Fleiss, 1979).

#### 2.2.1. The LLM Advantage in Formal Effectiveness

LLMs frequently surpass human performance in managing the cognitive load and executive errors associated with generating structurally flawless text (Gero et al., 2022; Yuan et al., 2022). Comparative studies of human and AI-generated narratives find that AI texts reliably receive higher scores for local and global coherence (Jakesch et al., 2023). This capacity allows LLMs to excel in writing tasks that demand high formality and low cognitive spontaneity. LLM output generally maintains a high degree of contextual relevance at the sentence and paragraph levels, reflecting their deep learning and efficient reproduction of optimal structural patterns from training data (Chang & Bergen, 2023). Even in creative narrative tasks, LLM outputs, though sometimes low in originality, are often more “formally correct” and “consistent” than human output (Lee et al., 2022). This aligns with the LLMs’ “fact-driven” and “rule-constrained” nature, enabling them to produce objectively and logically rigorous texts (Bender et al., 2021; Marcus & Davis, 2019). Dinu et al. (2025) found that humans excel in cross-domain associations, whereas LLMs demonstrate superior performance in logical rigor and the sheer volume of ideas. 

However, this relentless pursuit of effectiveness is often achieved at the expense of originality. LLM-generated narratives suffer from a significant lack of plot diversity, leading to homogenization. Doshi and Hauser (2024) found that despite the fluency of AI text, its Lexical Diversity (MTLD) is significantly lower than that of human expert writing.

#### 2.2.2. Human Uniqueness: Experience and Affect

While LLMs demonstrate mastery over formal effectiveness, research consensus consistently describes human creativity as “experience-driven” and fundamentally rooted in embodied cognition, personal memory, and affective processing (Glăveanu et al., 2013; Damasio, 1999). This grounding in subjective experience allows human authors to achieve feats currently beyond LLMs, including emotional depth and cross-domain conceptual association (Barsalou, 2008; Vervaeke et al., 2012).

Recent empirical research validates this distinction, particularly in the dimension of originality (Hutson, 2021; Mazzone & Elgammal, 2019). For instance, comparative studies on poetry generation have revealed a significant “affective gap”, where human-authored poems are consistently rated as more emotionally resonant, even when AI-generated poetry is structurally complex (Köbis & Mossink, 2021). This supports the argument that LLM output of emotion is a complex imitation of “affective language” rather than a representation of “psychological complexity” (Liu et al., 2023).

Similarly, in research on generating novel scientific hypotheses, LLMs excel at “local” novelty, but struggle to generate the “radical” novelty that defines human conceptual leaps (Park et al., 2023). This limitation is typically attributed to the LLM architecture, which is constrained by semantic relatedness within its training data, making it difficult to transcend conventional semantic categories (Mitchell & Krakauer, 2023).

#### 2.2.3. Contrasting Underlying Mechanisms of Creativity

Despite LLMs’ impressive achievements in creative output, consensus points to a fundamental divergence in the underlying mechanisms driving creativity. The selection of micro-linguistic features, such as lexical diversity and syntactic complexity, serves as a critical proxy for interpreting the mechanisms of creativity. For example, the average concreteness rating of all content words represents the mean concreteness of a text, based on norms from Brysbaert et al. (2014).

First, the driving force is fundamentally antithetical. Human output carries a unique subjective perspective, enabling authors to break through purely statistical associations and generate genuinely novel and meaningful content (Kaufman, 2016; Csikszentmihalyi, 1997). In contrast, LLM creativity is viewed as “fact-driven and rule-constrained” and reliant on the precise prediction of semantic correlations (Sutskever et al., 2014). Its novelty often manifests as statistically low-frequency combinations rather than a cognitive breakthrough (Floridi & Chiriatti, 2020).

Second, the reliance on structural optimization leads to patterning and diversity limitations. LLMs inherently face the risk of output homogenization (Messeri & Crockett, 2024). LLM stories tend to repeat or recombine highly frequent elements from the training data, resulting in a lack of plot diversity. While individual LLM outputs may seem creative, the collective output is highly homogeneous, contrasting sharply with human authorship (where, for example, story continuations rarely reproduce a Kafkaesque ending). 

Third, the emergence of LLMs does not eliminate individual differences in human creative performance (Karwowski et al., 2017). LLM assistance failed to eliminate the influence of human individual factors, such as creative ability and general intelligence, suggesting that LLMs function as ability amplifiers rather than eliminating individual differences in creativity (Zhou & Lee, 2024).

In summary, LLMs command formal effectiveness through statistical optimization, but they are structurally constrained, mechanistic, and lack the subjective grounding necessary for radical originality (Searle, 1980).

#### 2.2.4. Conflicts in Comparative Creative Performance

The most significant conflict in the field originates from contradictory findings when creative performance is aggregated across various tasks. No significant differences were found between human ideas and those generated by prompting specific GenAI models (Van Rooij & Biskjaer, 2025). High-performing humans still maintain a distinct edge in certain complex, high-demand creative tasks, such as those requiring conceptual leaps or profound subjective insight (Koivisto & Grassini, 2023). The observed performance asymmetry is fundamentally dependent on the specific cognitive resources demanded by the task, leading to a profound ambiguity regarding the nature of overall superiority.

1. The LLM Advantage in Divergent Tasks

In divergent thinking tasks that involve simple association and list generation, AI exhibits an overwhelming advantage in quantity. Empirical evidence from Koivisto and Grassini (2023) using the Alternate Uses Task (AUT) shows that AI chatbots surpass human participants’ average performance in both fluency (the quantity of ideas) and average originality. This is because the AUT essentially requires rapid retrieval from the semantic network (Kenett et al., 2014); the LLM’s vast parameterization allows it to instantly establish semantic connections (e.g., between “brick” and “paperweight”, “weapon”, or “artwork”) without the constraints of fatigue or cognitive load. However, the study also reveals a ceiling effect: the peak creative performance of top-performing humans still exceeds that of AI (Koivisto & Grassini, 2023). This suggests that AI raises the “creative floor”, but has yet to reach the “human ceiling” (Doshi & Hauser, 2024; Gero et al., 2022).

2. The Human Advantage in Complex Creative Tasks

When the task shifts to complex creative writing that requires long-range planning, sophisticated logical construction, and deep affective expression, the human advantage re-emerges (Yuan et al., 2022). The underlying reason is that creative writing demands not only lexical combination but also intentionality and theory of mind (Bruner, 1991; Mar & Oatley, 2008). Human authors pre-emptively anticipate reader response, embed foreshadowing, and construct character arcs with internal conflict. In contrast, LLMs, constrained by the context window and the probability prediction mechanism, tend to prematurely resolve conflicts or generate structurally homogeneous narratives lacking dynamic tension.

In summary, the key findings from the literature establish a clear task dependency: LLMs excel in quantity, average performance, efficiency, and structural conformance (effectiveness), particularly in divergent or propositional tasks. Conversely, humans maintain superiority in quality, depth, and genuine novelty (originality), particularly in complex tasks requiring embodied emotion and conceptual transformation.

### 2.3. Writing Assisted by LLMs: Augmentation or Diminishment

As Human–AI Collaboration (HAC) emerges as the new normal, scholarly attention has steadily shifted from competition to collaboration. The academic discussion broadly centers on two opposing yet intertwined perspectives: augmentation and diminishment.

#### 2.3.1. Augmentation: Raising the Floor

The augmentation thesis posits that LLMs raise the baseline quality of writing. Empirical studies demonstrate that Generative AI significantly boosts writing productivity, especially for individuals with lower writing competence. For these users, AI assistance acts as a powerful “scaffolding” mechanism, helping them overcome organizational barriers to produce work of average to standard quality—an effect termed “raising the floor” (Doshi & Hauser, 2024; Baron, 2023).

More broadly, GenAI serves as a powerful co-creator, automating initial idea generation and reducing cognitive fixation (Noy & Zhang, 2023; Zhou & Lee, 2024). LLMs provide a diverse range of starting points that ultimately enable humans to achieve higher levels of creative output. This suggests that the human role shifts from initial generation to strategic “curation”, “evaluation”, and “refinement” (Gero et al., 2022), where individual differences in creative ability and intelligence remain significant predictors of successful collaboration. 

#### 2.3.2. Diminishment: Homogenization and Cognitive Constraints

Conversely, the diminishment thesis warns that widespread reliance on GenAI may lead to a homogenization of creative output, a lack of true originality, and a resulting decline in human creative skills. This risk manifests through three primary mechanisms.

1. The Ceiling-Lowering Effect and Homogenization

While LLM-generated outputs may achieve high subjective scores on individual creative tasks, a deeper analysis often reveals a critical flaw: a lack of collective diversity. Research indicates that while AI assistance increases idea quantity, it may fail to improve—or even reduce—collective diversity, a phenomenon termed “Echoes in AI” (Xu et al., 2025; Peeperkorn et al., 2024). The mechanism of “Semantic Space Collapse” suggests that GenAI content converges on high-probability regions, leading to over-smoothed texts (Epstein et al., 2023).

2. Loss of Agency

The risk of loss of agency is also paramount. LLM assistance presents a double-edged sword: it effectively raises the baseline of acceptable output, but it simultaneously introduces a strong force for homogenization or illusion (Messeri & Crockett, 2024), potentially lowering the ceiling for true originality and reducing the creative agency of the human author.

### 2.4. Gaps in Prior Research and Objectives of the Present Study

Building upon the theoretical gaps identified in the preceding review—specifically the lack of micro-linguistic evidence and the ambiguity of task-dependent effects—the present study aims to address these fundamental limitations through a multi-stage comparative analysis.

#### 2.4.1. Gaps in Prior Research

1. Missing Task Typology Control

The central gap in comparative creativity research is the failure to systematically test and compare the performance of Large Language Models (LLMs) across contrasting task types. Mixed contexts obscure whether the structural power of LLMs yields a clear and universal advantage or whether human originality remains superior only in contexts demanding embodied cognition and complex affective integration. Without explicit control over task type (e.g., comparing low-level propositional vs. high-level creative tasks), research conclusions on comparative creativity lack generalizability and explanatory power.

2. Monolithic Evaluation Metrics

Most previous studies lacked the detailed explanatory power to distinguish between types of creative output. They have failed to systematically identify and analyze potential “creativity profiles” (e.g., “safe but mediocre”, or “novel but disorganized”). Neglecting the style distribution of creative performance prevents a complete understanding of how LLMs achieve high scores (e.g., through hyper-consistency) and where human creativity fails (e.g., due to disorganized novelty).

3. Black Box of Linguistic Mechanisms

Despite strong theoretical assertions regarding the underlying psychological drivers—that human creativity is subjective and experience-driven, while LLM creativity is statistical and rule-based (Boden, 2004)—these claims lack quantitative, explanatory validation. The inability to quantify these antithetical mechanisms severely hinders the field’s capacity to reveal the fundamental difference between human and artificial creative intelligence and limits the effective design of HAC systems.

#### 2.4.2. Research Objectives and Investigative Strategies

To resolve the empirical conflicts in the field, this study establishes a comprehensive framework designed to deconstruct the “Black Box” of artificial vs. human creativity. We move beyond simple score comparisons toward a tripartite investigation of creativity profiles and linguistic mechanisms. The following three objectives are central to this research:

Objective 1: Multi-dimensional cross-task validation:

Our primary goal was to determine if the purported human advantage in creativity is a universal trait or strictly task-dependent. To achieve this, we implemented a comparative strategy across two distinct writing phases: Phase 1 (foundational propositional tasks) and Phase 2 (high-demand creative fiction), allowing us to isolate how task complexity and cognitive demands shift the competitive edge between humans and LLMs.

Objective 2: Elucidating divergent linguistic mechanisms:

We aimed to identify the specific linguistic markers that serve as proxies for underlying cognitive processes. Our analytical strategy involved extracting 15 fine-grained features—such as lexical diversity and syntactic complexity—and employing Hierarchical Moderated Regression (HMR). This enabled us to empirically validate whether human creativity is indeed “experience-driven” while LLM creativity is “statistically optimized”.

Objective 3: Deconstructing the “collaboration trap” in human–AI interaction:

Given the emergence of Human–AI Collaboration (HAC) as a new norm, we sought to define the precise boundaries where AI assistance transitions from an “augmentor” to a “diminisher”. By comparing Human-Only, LLM-Only (GPT-5), and LLM-Assisted (GPT-2) groups, our experimental strategy tested human agency under suboptimal AI conditions to reveal the mechanisms of “semantic collapse” and “cognitive anchoring”.

By assessing both propositional and creative writing, this study moves beyond the “external frame of reference” that dominates traditional creativity research. We align with the emerging view that creative acts must be understood not just as the production of useful artifacts, but as satisfying cognitive engagements where the author’s internal intent meets the task’s structural demands (Abraham, 2025). This perspective allows for a more nuanced comparison between the mechanistic optimization of LLMs and the intent-driven originality of human authors.

## 3. Materials and Methods

### 3.1. Participants and Materials

This study employed a two-stage, mixed-methods experimental design to compare the creativity and linguistic characteristics of texts generated by human writers, Large Language Models (LLMs), and human writers assisted by an LLM. The analysis categorized the resulting texts into three distinct author types:

Human-Only group (H): Texts composed by human participants without any LLM assistance.
LLM-Only group (L): Texts generated entirely by the designated LLM via API.
LLM-Assisted group (A): Texts composed by human participants who utilized an LLM as an integrated tool during the writing process.

#### 3.1.1. Human Participants

Phase 1 (propositional tasks): Participants were 8th-grade students recruited from China. Each student was randomly assigned one of three prompts and composed a single essay without external aid.

Phase 2 (creative task): Participants were 50 sophomore undergraduate students in China, divided into two equal groups (N = 25 each) for the Human-Only (H) and LLM-Assisted (A) conditions.

#### 3.1.2. LLM Generation

LLM-Only group (L): Texts were generated by GPT-5 (the latest commercial model available at the time of data collection). For all tasks (Phase 1 and Phase 2), the model was queried via API to generate the target number of texts per prompt. 

LLM-Assisted group (A) Tool: For the human participants in the Phase 2 LLM-Assisted group, the foundational model used for assistance was GPT-2 due to the specific technical constraints of the early work environment.

#### 3.1.3. Writing Tasks and Corpus

##### Phase 1: Propositional Writing Tasks

Phase 1 involved propositional writing tasks (P1, P2, and P3). Participants and LLMs were tasked with composing short essays, adhering to a minimum word count of 400 Chinese characters, with no restrictions placed on genre or writing style. The three prompts (P1, P2, and P3) were “Company is the best gift”, “If I could do it again”, and “I forgot ________”, requiring the author to complete the title before writing.

##### Phase 2: Creative Writing Task

Phase 2 focused on a Creative Writing Task (CT), designed to probe sophisticated ideational expansion and the utility of LLM assistance in a structured workshop setting. This task required the creation of a piece of speculative fiction centered on the theme of future work in the year 2040. The composition had to incorporate two specific, pre-designed virtual background contexts: “The Age of AI Involution” and “Data Generators as Workers”. The two types of writing task prompts are specified in Appendix A.

The distinct nature of the Phase 2 task (from propositional to high-level creative fiction) was chosen to explore whether the impact of LLMs differed based on the openness and complexity of the creative domain. 

The initial collected texts were subjected to a data cleaning process. Texts were excluded if they were deemed invalid (blank answers, sentences copied from prompts, text written in a non-Chinese language, and off-topic) or unrecognized slang (stacked idioms; slang; or unknown symbols that do not constitute smooth and meaningful sentences, often without punctuation). The final, cleaned sample size distribution across the three author types and the four writing tasks is presented in Table 1.

While the human sample was localized, several factors ensured its results are statistically indicative. First, the total corpus of 1296 texts provided high statistical power for the Hierarchical Moderated Regression (HMR) and ANOVA models, exceeding common requirements for detecting medium-to-large effect sizes. Second, the recruitment of 8th-grade students (Phase 1) and university sophomores (Phase 2) was strategically chosen to align task complexity with developmental appropriateness, creating a robust benchmark for “foundational” vs. “high-demand” writing. The inclusion of nearly 700 LLM-generated texts further stabilized the comparative baseline, ensuring that the human–AI performance gap was measured against a statistically significant volume of AI output.

### 3.2. Experimental Procedure

To ensure transparency and reproducibility, the study followed a linear four-step experimental workflow:

Step 1: Task assignment. Participants and LLMs were assigned to propositional tasks (Phase 1) or a creative task (Phase 2).
Step 2: Text generation. Texts were produced across three author types: Human-Only, LLM-Only (GPT-5), and LLM-Assisted (utilizing GPT-2 as a suboptimal tool).
Step 3: Data preprocessing. A rigorous cleaning protocol excluded blank, off-topic, or non-Chinese language responses, resulting in a final corpus.
Step 4: Expert rating. Five trained raters independently evaluated each text based on the dual-dimensional rubric of originality and effectiveness.

### 3.3. Creativity Assessment and Rating Protocol

#### 3.3.1. Scoring Rubric

The study’s primary dependent variables were the two core dimensions of creativity: Originality (O) and Effectiveness (E), measured via expert human ratings on a 5-point Likert scale (1 = lowest, 5 = highest).

Originality (O): This measured the degree of novelty, uniqueness, and imaginative quality of the text. It was calculated as the aggregate mean of sub-scores related to Inspiration.
Effectiveness (E): This measured the degree of functional appropriateness, execution quality, and successful communication of the intended message.

The rubric was guided by a theoretical framework developed by a panel of five subject-matter experts, including experienced teachers, psychometricians, and language testing specialists. To construct the scoring rubric, a small random sample (25%) of essays from each writing task was extracted and rigorously analyzed by the expert panel. Following multiple rounds of discussion, consensus building, and revision based on the initial samples, a 5-point analytic scoring rubric was generated (see Appendix B). Crucially, the rubric was designed as a norm-referenced tool, emphasizing the degree to which an essay’s originality and effectiveness demonstrated creativity relative to other essays generated under the exact same writing prompt. The 5-point scale ranged from 1 (lowest level of creativity) to 5 (highest level of creativity).

#### 3.3.2. Rater Training and Scoring

Five raters (two male and three female graduate students majoring in psychology and linguistics) were recruited and trained to score the essays using the established 5-point rubric. The training followed a strict calibration protocol to minimize idiosyncratic scoring bias:

Initial Training: Raters were thoroughly instructed on the theoretical definitions of originality and effectiveness and the operational descriptions for each level of the 5-point scale.
Calibration Session: Prior to formal scoring, 15% of the total essay sample was independently evaluated by all five raters.
Consensus Meeting: Notable discrepancies and differences in opinion regarding the trial scoring were identified and discussed in detail to refine the raters’ shared understanding and application of the rubric descriptors, ensuring a consensus on the scoring standards was reached.

After the calibration phase, the raters independently scored all remaining essays in a pseudo-random order. Each essay received five independent scores for both the originality and effectiveness dimensions.

#### 3.3.3. Reliability Assessment

Inter-rater reliability (IRR) for the two dimensions was assessed using the ratings provided by five human raters (k = 5). We report two highly complementary statistics to demonstrate the robustness and internal consistency of the aggregated scores: the ICC and Cronbach’s α. Since our main analysis relies on the mean score across all five raters, the ICC(A, k) directly measures the reliability of this aggregated score. Cronbach’s α complements this by demonstrating the scale’s internal consistency (i.e., the extent to which the raters are consistent in their application of the rating scale). 

The results indicate that inter-rater reliability was generally high across all tasks and dimensions. For originality, the ICC(A, k) values ranged from 0.70 to 0.87 (M = 0.79), and Cronbach’s α values ranged from 0.71 to 0.88 (M = 0.81). For effectiveness, the ICC(A, k) values ranged from 0.70 to 0.84 (M = 0.77), and Cronbach’s α values ranged from 0.75 to 0.92 (M = 0.82). According to conventional psychometric criteria (ICC ≥ 0.75 and α ≥ 0.80 indicating good reliability), these results demonstrate satisfactory stability and agreement among raters. Consequently, the mean score across the five raters was used as the final writing score for subsequent predictive modeling and statistical analyses.

### 3.4. Computational Linguistic Feature Extraction

After identifying different quality features through cluster analysis, this stage of analysis aimed to reveal the underlying language mechanisms driving these features and explain the observed differences in creativity scores. We investigated how fine-grained text features predict the two core dimensions of creativity—originality and validity—and, crucially, whether these predictive relationships are moderated by the author type. This explanatory model provides a key connection between descriptive group differences and emerging creativity profiles, from “what” to “how” and “why”. The methodological transparency of our feature selection rests on a two-stage data-driven validation: (1) Candidate Selection: an initial pool of over 50 features was computed using a custom Chinese analysis tool; (2) Predictive Validation: correlation analysis was performed against creativity scores, and only the 15 features demonstrating significant predictive validity were retained for the final regression models.

#### 3.4.1. Feature Selection and Extraction

To build predictive models, we employed a hybrid approach, combining theory-driven selection with data-driven validation, to identify a robust set of 15 computational linguistic features. To provide a clear and reproducible overview of these features, their operational definitions, and their theoretical links to creativity, we present them in Table 2. As shown, these features were chosen for their potential to proxy underlying cognitive processes, such as cognitive load (e.g., lexical diversity), mental imagery generation (e.g., concreteness), and structural complexity (e.g., dependency distance), which are critical for the production of creative text. All features were extracted automatically from each text using a custom Chinese text analysis tool. The final selection of these 15 features was empirically validated. A larger pool of over 50 candidate features was initially computed. We then performed correlation analysis against the creativity scores (originality and effectiveness). Only features demonstrating a statistically significant correlation (*p* < 0.05) with at least one creativity dimension in the full sample were retained, ensuring their predictive validity.

#### 3.4.2. Modeling and Rationale Analysis

To ensure the robustness of the regression models, all continuous predictor and dependent variables underwent a two-step preprocessing procedure: (1) Winsorization: To mitigate the influence of extreme outliers, all feature variables were winsorized at the 1st and 99th percentiles. (2) Z-Standardization: All variables were converted to z-scores (mean = 0, SD = 1). This standardization facilitates the direct comparison of regression coefficients (effect sizes) and improves model interpretability and convergence. We employed hierarchical ordinary least squares (OLS) regression models for each creativity dimension (originality and effectiveness). The primary goal was to model the interaction between author type and each linguistic feature. Phase 1 is demographically and cognitively homogeneous, allowing us to isolate the effect of author type (human vs. LLM) on the relationship between linguistic features and creativity within a consistent task context. The model is specified as follows:

Creativity_Score = β_0_ + β_1_*AuthorType + Σ(β_i_*Feature_i_) + Σ(γ_i_*(AuthorType × Feature_i_)) + ε

where

Creativity_Score is the mean rater score for either originality or effectiveness;
AuthorType is a dummy variable (0 = Human-Only, 1 = LLM-Only);
Feature_i_ represents one of the 15 standardized linguistic features;
γ_i_ is the coefficient for the interaction term, the primary parameter of interest.

The model was built hierarchically:

Model 1 (Main Effects) included AuthorType and all 15 Feature_i_ main effects.
Model 2 (Full Model) added the 15 interaction terms (AuthorType × Feature_i_) to Model 1 to test for moderation.

To ensure the validity of our inferences, we checked the model assumptions: (1) Multicollinearity: This was assessed using the Variance Inflation Factor (VIF). All features in the models demonstrated VIF values well below the conventional threshold of 10 (all VIF < 5.96), indicating no problematic multicollinearity. (2) Residual Diagnostics: The normality of residuals was confirmed using the Shapiro–Wilk test (*p* > 0.05), and homoscedasticity was confirmed using the Breusch–Pagan test (*p* > 0.05) for all final models. Given the multiple interaction terms tested (k = 15), we controlled the False Discovery Rate (FDR) using the Benjamini–Hochberg procedure. For the interaction terms, an FDR-adjusted *p*-value of less than 0.10 was considered statistically significant. For the main effects, a standard *p* < 0.05 threshold was used. For all significant interactions, simple slope analyses were conducted.

### 3.5. Statistical Analysis Strategy

In the interest of rigorous scientific transparency, we address two key constraints inherent in the two-phase research design, detailing their implications and the analytical strategies used to mitigate potential confounding effects.

#### 3.5.1. Constraint 1: Cross-Sectional Cohort Differences

Constraint: Phase 1 participants (propositional tasks) were 8th-grade students, while Phase 2 participants (creative writing task) were university sophomores. This cross-sectional design means that the two task groups differed significantly in cognitive maturity, writing proficiency, and content knowledge, which are known confounding factors in writing performance studies. Phase 1 tasks (P1, P2, and P3) represent standard expository and narrative writing skills, appropriate for measuring foundational writing creativity in secondary education. Phase 2 (Creative Task, CT) demands higher-order creative thinking, speculative world-building, and integration of complex social/technological themes, which is cognitively more suitable for advanced university students.

The study’s core research questions focus on within-task comparisons, rather than direct cross-task comparisons between P1 and CT. Any comparison between the propositional and creative tasks (e.g., the interaction effect between task type and author type) is interpreted as the difference between a foundational writing task and a high-demand creative task, acknowledging the inherent cohort difference as an intrinsic part of task difficulty and the required skill set. We explicitly acknowledge that task category and participant cohort are not fully orthogonalized in this design. Consequently, any observed differences between Phase 1 and Phase 2 are interpreted as contextual variations, reflecting a bundle of task complexity, openness, and the developmental appropriateness of the writers, rather than being attributed solely to task type as an isolated causal factor.

#### 3.5.2. Constraint 2: Inconsistent LLMs Across Groups

The LLM-Assisted group (A) utilized GPT-2 as the integrated tool for human participants during the creative writing workshop, whereas the LLM-Only group (L) for both phases was generated using the state-of-the-art GPT-5. This inconsistency introduced a non-negligible difference in tool quality. Technical and Historical Justification: The use of GPT-2 in the LLM-Assisted workshop setting (Phase 2) was primarily necessitated by technical constraints, accessibility, and stability limitations inherent to early large-scale educational workshops at the time of data collection.

This is a common and necessary compromise in applied educational research. This model selection was reframed as an analytical design to test two distinct hypotheses: LLM-Only (L) with GPT-5 serves as the upper bound benchmark of current (state-of-the-art) LLM writing performance. It represents the ideal performance level that a top-tier generative model can achieve independently. LLM-Assisted (A) with GPT-2 tested the effect of human agency under suboptimal conditions. The key question is as follows: Can human collaboration and agency, using a structured creative process, effectively leverage an older model to achieve superior creative outcomes compared to the Human-Only group (H), and how close can they come to the GPT-5 benchmark? 

### 3.6. Data Analysis Strategy

#### 3.6.1. Comparative Analysis

To test for significant differences in the core dimensions of perceived text creativity—originality and effectiveness—across different author types and task types, a two-stage approach using analysis of variance (ANOVA) was implemented. The overall analysis strategy was designed to align with the core research questions: first, testing for global differences, and second, identifying specific group differences within each task context.

#### 3.6.2. Unsupervised Clustering Analysis

To uncover latent, evaluator-perceived patterns in textual quality, we employed an unsupervised clustering analysis. This technique utilized the two continuous dimensions of perceived quality—originality and effectiveness—as inputs, allowing us to move beyond conventional mean-level comparisons and identify distinct creativity profiles inherent in the pooled rating data.

Given the bivariate, continuous nature of the input space, we applied k-means clustering. This partitioning algorithm minimizes the within-cluster sum of squared Euclidean distances to maximize the homogeneity within each resulting cluster.

The optimal number of clusters (k) was determined through a consensus of internal validation metrics, including the silhouette coefficient, the Calinski–Harabasz Index, and the elbow method, evaluating solutions from k = 2 to k = 5. The four-cluster solution (k = 4) was ultimately selected. The average silhouette score was 0.38. While this indicates a modest structure, it supports the exploratory identification of emergent writing patterns across the corpus. This decision was based not only on yielding the silhouette score but, critically, on its ability to produce theoretically meaningful and educationally interpretable profiles that clearly differentiate quality types.

To ensure the identified profiles reflected a consistent application of the rating standards across tasks, clustering was conducted on the full sample of evaluation scores (N = 1498), encompassing texts from both the propositional and creative writing tasks. The resulting four clusters were subsequently labeled based on their respective mean scores for originality and effectiveness.

#### 3.6.3. Hierarchical Moderated Regression (HMR) for Mechanism Exploration

This stage of the analysis was designed to move beyond descriptive group differences and identify the underlying linguistic mechanisms that predict perceived creativity, examining whether these relationships are contingent upon the authoring agent. Specifically, we employed Hierarchical Moderated Regression (HMR) to test the moderating effect of author type on the predictive power of the computational linguistic features.

##### Model Specification

Two separate full factorial HMR models were conducted—one for the mean originality score and one for the mean effectiveness score. These analyses were performed separately for the propositional tasks and the creative task. The general form of the HMR model used for each of the features was structured hierarchically.

##### Procedural Steps

The linguistic features were z-transformed prior to analysis, and the continuous predictors were mean-centered before calculating the interaction term to mitigate multicollinearity concerns. Step 1 (Main Effects): The independent variable (X) and the moderator (M) were entered into the model to establish their main effects (β1, β2). Step 2 (Interaction Test): The interaction term (X • M) was added. The significance of β3 and the corresponding change in R-squared (∆R^2^) indicated whether the predictive relationship between the linguistic feature and creativity was significantly moderated by the author type.

This approach allowed for the identification of specific linguistic mechanisms where human and LLM authors diverge in their path to achieving (or failing to achieve) high creative ratings.

#### 3.6.4. Specific Analysis of Human–AI Collaboration

This analysis specifically targeted the creative writing task, investigating the impact and mechanisms of the LLM-Assisted process by comparing the three distinct author groups: Human-Only (H, N = 25), LLM-Assisted (A, N = 25), and LLM-Only (L, N = 79). This comparative analysis aimed to understand the utility of human collaboration, particularly given the technical constraint of using the GPT-2 model for assistance. 

##### Comparative Assessment of Creativity Scores

To test for differences in originality and effectiveness across the three groups, two separate one-way analyses of variance were performed (one for each dimension). Following any statistically significant ANOVA result, Tukey’s Honestly Significant Difference (HSD) post hoc test was used to identify all pairwise differences among the three author types, controlling for the familywise error rate.

##### Linguistic Mechanism Comparison

To reveal the underlying stylistic and structural influence of the GPT-2 tool on the human writers, a series of one-way ANOVA models was conducted on the 14 features (as defined in Section 3.4.1). The hypothesis was that this analysis tested whether the linguistic profile of the LLM-Assisted group shifted towards the LLM-Only group (e.g., higher ParaSim, Difficulty) or retained the characteristics of the Human-Only group (e.g., First-Person Singular). Given the unequal group sizes (N = 25, N = 25, N = 79), Tukey’s HSD post hoc test was used for all significant ANOVA results to ensure robust inference of specific group differences, particularly distinguishing the HAC style (A) from the two baseline conditions (H and L).

## 4. Results

### 4.1. Comparative Performance: Task-Dependent Asymmetry

To evaluate authorial and task impacts on perceived writing creativity, two separate two-way full factorial ANOVAs were conducted, using originality and effectiveness as the dependent variables. The independent variables were author type (Human-Only vs. LLM-Only) and task category (propositional tasks vs. creative task). The initial ANOVA analysis revealed two pivotal findings: (1) originality is context-dependent, with humans outperforming LLMs only in high-demand creative tasks (*p* < 0.001), but matching them in propositional tasks; (2) LLMs govern effectiveness universally, maintaining a significant and substantial advantage across all task types (Partial η^2^ = 0.208). These results immediately demonstrate that while LLMs raise the performance floor, human superiority remains confined to complex, intent-driven domains.

#### 4.1.1. Global 2 (Author Type) × 2 (Task Category) ANOVA Results

The results of the overall two-way ANOVA reveal a complex pattern of main and interaction effects (see Table 3).

**Figure 1:**
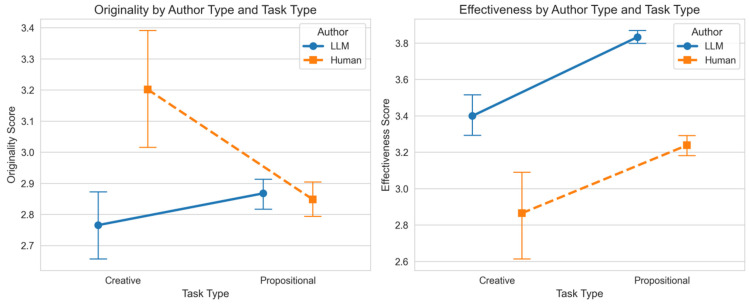
Scores of originality and effectiveness by author type (human vs. LLM) and task type (creative vs. propositional). This figure presents the interaction effects between authorship and task type on two dimensions of writing creativity: originality (**left panel**) and effectiveness (**right panel**).

For originality, the main effect of author type did not reach statistical significance. Critically, a significant two-way interaction effect was found between author type and task category. This indicates that the difference in originality between human and LLM-generated texts is dependent on the type of task they are completing; see Figure 1 (left).

For effectiveness, significant main effects were found for author type and task category. Specifically, the LLM-Only group scored significantly higher on effectiveness than the Human-Only group, and the propositional tasks generally yielded higher effectiveness scores than the creative task. Crucially, the interaction effect was not significant, suggesting that the LLM’s advantage in effectiveness is consistent across both task categories; see Figure 1 (right).

#### 4.1.2. Follow-Up Task-Specific Comparisons (*t*-Tests)

Given the significant interaction effect for originality and the significant main effects for effectiveness, independent samples *t*-tests were conducted to compare the scores of the author types within each of the four individual writing tasks (P1, P2, P3, and CT), with the results presented in Table 4.

The results indicate a critical qualitative difference in how the overall task environment—encompassing both constraints and participant maturity—affected the comparative advantage in originality. The Human-Only group achieved significantly higher originality only in the Creative Task (CT). This is consistent with theories suggesting that human creativity excels under high-demand tasks that require complex, novel conceptual blending, a domain where current LLMs often resort to predictable, highly probable sequence generation.

However, despite the presence of an open component, the semi-propositional task P3 (requiring authors to complete the title) did not result in a significant human advantage over the LLM-Only group. This non-significant finding for P3, along with the other two fully propositional tasks (P1, P2), suggests that the mere allowance of textual input freedom (e.g., completing a title) is insufficient to activate the unique human advantage in originality.

The results indicate a critical qualitative difference in how the overall task environment—encompassing both constraints and participant maturity—affected the comparative advantage in originality. Since P1, P2, and P3 are fundamentally propositional tasks (focused on argumentation, explanation, or realistic narrative), the rater’s perception of “originality” likely shifted from pure conceptual novelty (as in CT) to novel combination of facts or rhetorical effectiveness. In this context, the LLM’s ability to rapidly synthesize vast training data into logically coherent and varied structures allows it to match human performance. The minor addition of title completion in P3 did not shift the fundamental cognitive demands of the task enough to differentiate human versus AI performance in novelty.

The observed interaction indicates that the relative advantage of human versus LLM authorship is contingent upon specific task contexts. Specifically, the human advantage in originality emerged in the high-demand creative task supported by cognitively mature university writers, whereas no such advantage was found in the foundational propositional tasks completed by younger students.

Across all four tasks, the LLM-Only group demonstrated a statistically significant and substantially larger mean score for effectiveness compared to the Human-Only group. The effect sizes observed for effectiveness were extraordinarily large, ranging from d = −0.84 in the creative task to a monumental effect of d = −1.18 in the propositional tasks. These values indicate that LLMs do not merely perform better; they fundamentally govern execution quality, establishing a new benchmark for structural and rhetorical competence that significantly transcends typical human performance in these contexts.

### 4.2. Exploratory Creativity Profiles

The k-means clustering identifies four distinct stylistic patterns: Ideal (High O, High E), Plain (Low O, Mid-E), Safe (Low O, High E), and Moderate (Mid-O, Low E).

#### 4.2.1. Identified Creativity Profiles Through Cluster Analysis

K-means clustering suggested four exploratory writing creativity profiles, based on the dimensions of originality and effectiveness, confirming the theoretical validity of the four-cluster solution. These clusters were subsequently labeled based on the magnitude of their mean scores (see Table 5). The entire corpus was partitioned into four distinct creative styles based on the distribution of originality (O) and effectiveness (E) scores.

The four exploratory profiles identified in this study, listed by cluster index and descriptive label, are as follows:

Cluster 0: High originality, high effectiveness (Ideal): This profile represents the optimal creativity, characterized by high mean scores on both originality and effectiveness. This cluster constituted 22.61% of the total analyzed corpus.
Cluster 1: Low originality, middle effectiveness (Plain): This profile was characterized by the lowest mean originality score and mid-level effectiveness. Texts here are generally routine and predictable, though they possess a moderate degree of competence.
Cluster 2: Low originality, high effectiveness (Safe): This was the largest cluster, which is defined by low-level originality but relatively high effectiveness. Texts in this profile are well-executed and competent but lack innovative or unique elements.
Cluster 3: Middle originality, low effectiveness (Moderate): This profile showed a mixed pattern: mid-level originality but the lowest effectiveness score among the four clusters. This suggests texts that attempted unique ideas but struggled significantly in execution and coherence.

#### 4.2.2. Distribution of Creativity Profiles by Author Type

**Figure 2:**
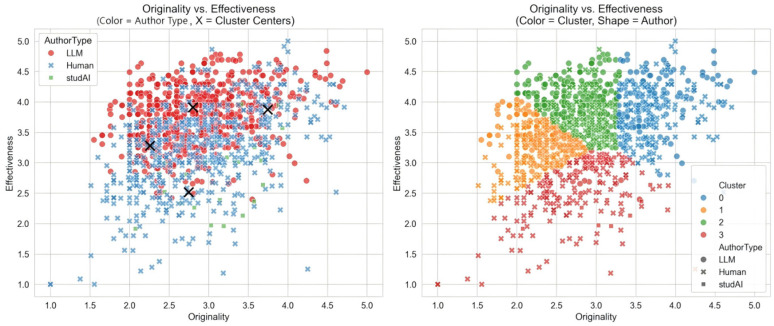
Distribution of texts in the originality–effectiveness space by author type (**left panel**) and cluster membership (**right panel**). The left panel displays individual text ratings on originality and effectiveness, colored by author type: red circles for LLM-generated texts, blue crosses for human-authored texts, and green squares for studAI texts. Black “X” marks indicate cluster centroids from k-means clustering based on these two dimensions. The right panel shows the same data with points color-coded by cluster membership.

The two-dimensional plot in Figure 2 (left) visually represents the mean location of the four clusters, illustrating the clear separation achieved by the k-means algorithm.

A χ^2^ test of independence was performed to examine the relationship between the categorical creativity profile membership and the author type (human vs. LLM). The results indicate a highly significant association between the two variables, χ^2^ = 191.32, *p* < 0.001. Further analysis of the conditional column percentages (Table 6) reveals the stark distribution differences.

As shown in Table 6, a striking distribution emerges: LLMs dominate the “Safe Style” (73.38%), while humans are predominantly found in the “Plain Style” (84.92%). This confirms that LLMs prioritize risk-averse competence, whereas average human writers struggle with execution.

Low O, high E (Safe) texts were overwhelmingly dominated by LLM-generated content, indicating LLMs are highly proficient at producing effective, though less original, texts; low O, mid-E (Plain) texts were primarily attributed to human authors, suggesting human writers, particularly in this sample, frequently produced texts that lacked both high originality and high effectiveness; high O, high E (Ideal) and mid-O, low E (Moderate) profiles showed a near-equal distribution between human and LLM authors, indicating that both author types are capable of achieving these quality patterns, although the profile suggests LLMs are slightly more common in texts with higher originality but lower effectiveness.

### 4.3. Mechanism Exploration: The Moderating Role of Authorship on Linguistic Predictors

Following the identification of distinct creative profiles, this section reports the results of the Hierarchical Moderated Regression (HMR) analysis. The primary goal was to test whether the predictive relationship between computational linguistic features and the creativity scores is significantly moderated by author type.

#### 4.3.1. Overview of Moderation Effects

As shown in Table 7, In both the propositional and creative tasks, the author type was found to be a significant moderator (*p* < 0.05) in 11 of the 56 models tested, demonstrating that the underlying linguistic mechanisms driving creativity differ fundamentally between human and LLM authors.

#### 4.3.2. Linguistic Mechanisms in Propositional Tasks

**Figure 3:**
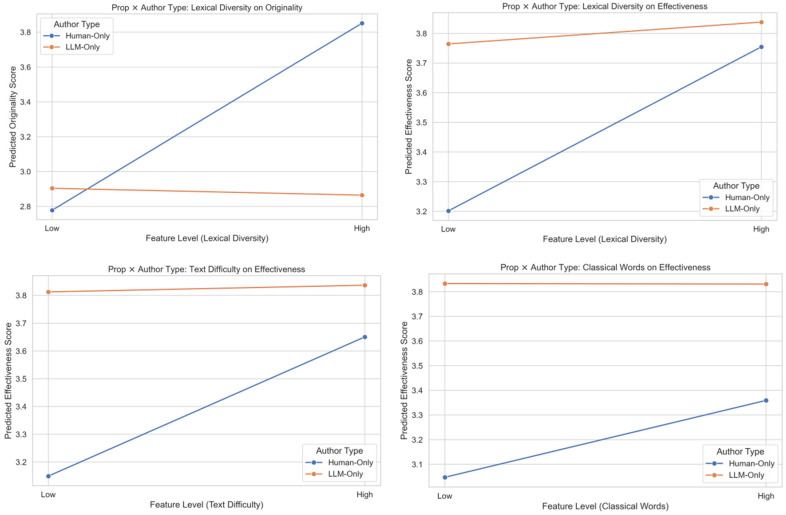
Interaction effects of the linguistic feature and author type on creativity in propositional tasks. The plot illustrates the significant moderating effect of author type (human vs. LLM). The x-axis represents the level of the linguistic feature (predicted at one standard deviation below and above the mean), and the lines represent the two author groups. The divergence in the slope between the LLM-Only group (orange line) and the Human-Only group (blue line) confirms that the predictive relationship between the linguistic feature and creativity is contingent upon the authoring agent.

As shown in Figure 3, in the foundational propositional writing tasks, interactions were primarily concentrated on features related to lexical complexity and fluency.

Originality Mechanism: The strongest interaction observed was for MTLD. As visualized in the corresponding interaction, higher lexical diversity positively predicted originality for Human-Only texts, but this positive relationship was canceled out or reversed for LLM-Only texts. This indicates that LLM’s attempt at synthesizing varied vocabulary did not translate into a perceived creative advantage in the propositional context.

Effectiveness Mechanism: Multiple features significantly moderated effectiveness. The negative interactions for difficulty and MTLD suggest that while LLM’s default output is highly effective, pushing it toward higher linguistic complexity or diversity (which correlates with higher human scores) is detrimental to its perceived competence relative to the human baseline. Conversely, the negative interaction for Classical Words indicates that the LLM was highly effective at deploying specific stylistic features and almost completely offset the negative impact of high lexical diversity on effectiveness. This demonstrates the inherent robustness of LLMs in handling complex linguistic features and their ability to stably integrate high lexical diversity without sacrificing fluency. However, the utility of this feature was diminished (less positive slope) compared to when human authors successfully employed it.

#### 4.3.3. Linguistic Mechanisms in the Creative Task

**Figure 4:**
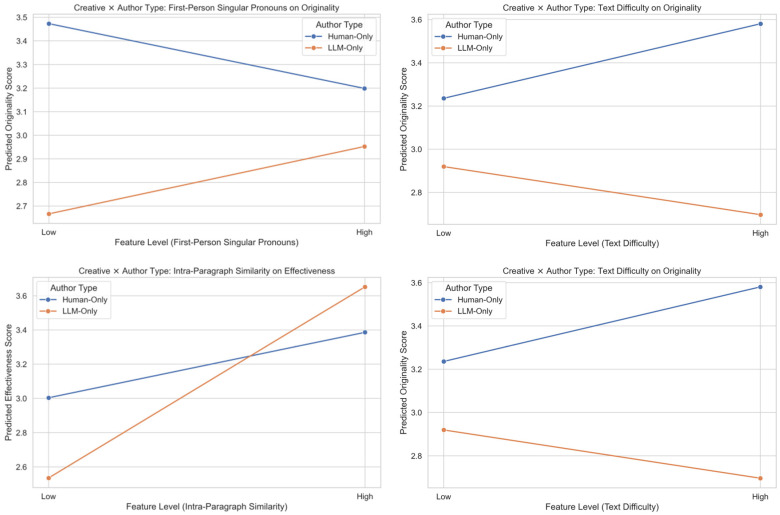
Interaction effects of the linguistic feature and author type on creativity in the creative task. The plot illustrates the significant moderating effect of author type (human vs. LLM). The x-axis represents the level of the linguistic feature (predicted at one standard deviation below and above the mean), and the lines represent the two author groups. The divergence in the slope between the LLM-Only group (orange line) and the Human-Only group (blue line) confirms that the predictive relationship between the linguistic feature and creativity is contingent upon the authoring agent.

As shown in Figure 4, in the high-demand creative task, the interactions shifted focus toward deep semantic and structural coherence.

Originality Mechanism: The highest ∆R^2^ in this context came from First-Person Singular (∆R^2^ = 0.0469, *p* = 0.019). This implies that the impact of subjective narrative language was highly dependent on authorship. Given that human texts excelled in originality in this task, this finding suggests that the unique advantage of human creative writing may be tied to the authentic, subjective use of personal voice. The negative Difficulty interaction (β Interaction = −0.001, *p* = 0.040) mirrors the propositional tasks, indicating that for LLMs, increasing textual difficulty does not pay the same originality dividends as it does for humans.

Effectiveness Mechanism: The Intra-Paragraph Similarity (ParaSim) interaction (β Interaction = 2.087, *p* = 0.008) was highly significant. This large positive interaction indicates that LLM’s effectiveness relies heavily on maximizing internal thematic consistency; the predictive slope of coherence on effectiveness is much steeper for LLM texts than for human texts. This suggests that LLM’s advantage in competence stems from a narrower, coherence-driven mechanism, as shown in the corresponding interaction figure.

Collectively, these HMR results reveal a fundamental divergence in the mechanisms of creativity: human creativity benefits from complexity and narrative depth, while LLM creativity is primarily driven by formulaic fluency and maximal coherence, which, when pushed to extremes, can lead to diminishing returns relative to human performance.

### 4.4. Style-Specific Linguistic Mechanisms of Human–AI Collaboration

Building on the primary H versus L comparisons, this section presents the results of the specific analysis for the Creative Task (CT), including the LLM-Assisted group (A). To precisely characterize the unique linguistic fingerprint of the LLM-Assisted group (A) and its relationship to the desired creative outcome, we analyzed how the three author types distributed across the four identified creativity profiles and how their linguistic features varied within these profiles.

#### 4.4.1. Comparative Assessment of Creativity Scores and Profile Distribution

As shown in Table 8, The distribution of the three author types across the four creative styles confirms that LLM influence fundamentally alters creative strategy in the CT. The results show that the LLM-Only group was concentrated heavily in the Safe and Plain profiles. This confirms that the state-of-the-art LLM (GPT-5) prioritizes generating texts that are either maximally competent (Safe: high E) or routinely satisfactory (Plain: mid-E). Both human-involved groups concentrated significantly in the Moderate style. This style is characterized by mid originality but low effectiveness. This pattern suggests that when attempting high-level speculative fiction, human writers (with or without GPT-2 assistance) prioritize novelty and idea generation over flawless execution.

The ANOVA results reveal distinct patterns for the two creativity dimensions and are summarized in Table 9.

Originality: Although the Human-Only group exhibited the highest numerical mean, the overall ANOVA was not statistically significant. This indicates that the addition of LLM assistance and the use of the high-end LLM did not significantly alter the perceived novelty among the three authoring modes.

Effectiveness: The results were highly significant. Post hoc analysis confirmed that the LLM-Only group scored significantly higher on effectiveness than both the Human-Only and LLM-Assisted groups. Crucially, the LLM-Assisted group exhibited the lowest numerical mean and showed no significant difference from the Human-Only group, suggesting that collaboration with the GPT-2 tool failed to provide an advantage.

#### 4.4.2. The Linguistic Mechanism of the Human–AI Collaboration Trap

To reveal the underlying stylistic and structural influence of the GPT-2 tool on human writers, a series of one-way ANOVAs was conducted on the linguistic features across the three groups. This analysis sought to determine if the LLM-Assisted group’s linguistic profile shifted toward the high-coherence, high-complexity LLM-Only group or retained the characteristics of the Human-Only group. The analysis reveals two critical findings regarding the linguistic fingerprint of the LLM-Assisted group (A), as summarized in Table 10:

Shift towards LLM-like fluency: The LLM-Assisted group’s texts were significantly pushed toward the LLM-Only style in key competence metrics. (1) Intra-Paragraph Similarity (ParaSim): LLM-Only texts showed the highest coherence, followed by the LLM-Assisted texts, with Human-Only texts showing nearly zero similarity. The LLM influence, even from GPT-2, immediately pushed the text toward maximizing structural consistency (higher ParaSim). (2) Complexity (Difficulty and Classical Words): LLM-Assisted texts occupied the intermediate ground on these metrics, indicating that GPT-2 assistance significantly increased the linguistic complexity and difficulty of the human outputs, moving them away from the simplicity of the Human-Only group.

Preservation of Human Subjectivity and Syntactic Inefficiency: Despite adopting LLM-like complexity, the collaboration maintained key human stylistic features. (1) First-Person Singular: Both the Human-Only and LLM-Assisted groups used significantly more first-person language than the LLM-Only group. The LLM-Assisted group had the highest numerical mean. This suggests that the human writers leveraged AI fluency while maintaining or increasing their own subjective narrative input.

Effectiveness Failure: The lack of effectiveness improvement alongside this mixed linguistic outcome suggests a “collaboration trap”. The LLM-Assisted group inherited the AI’s tendency toward structural complexity (Difficulty) but failed to translate this into improved coherence or execution (no gain in effectiveness), indicating that the suboptimal GPT-2 model was unable to be a fully competent partner.

#### 4.4.3. Author Type × Style Interaction on Linguistic Feature

A series of two-way ANOVAs revealed significant author type × style interactions for four language features: Paragraph Similarity (Adj.), Lexical Diversity (MTLD), Text Difficulty, and Words Per Sentence (see Table 11). Subsequent simple effects analyses (Tukey-adjusted) uncovered systematic differences, particularly within the Moderate and Plain styles. Partial eta squared (η^2^_p_) was reported as the effect size metric. 

**Figure 5:**
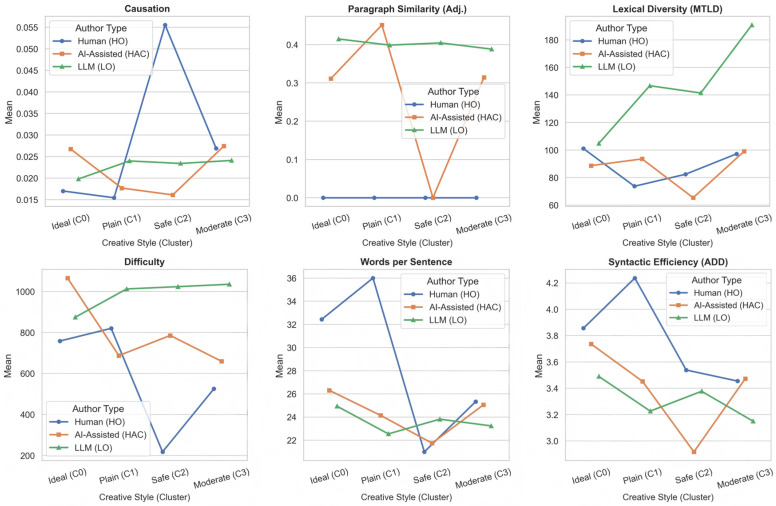
Interaction effects of author type and creative style on linguistic features. This figure presents the interaction effects between author type and creative profile on three key linguistic features.

The existence of these interactions confirms that the linguistic mechanisms employed by the authors are contingent upon the creative goal they are pursuing. Simple effects analysis (Tukey-adjusted) revealed systematic linguistic divergence between the author groups, particularly within the moderate (mid-O, low E) profile where human involvement was highest. Figure 5 presents the interaction effects of author type and creative style on linguistic features.

Moderate Profile (mid-originality, low effectiveness): This was the most populated cluster overall. LLM-generated texts exhibited substantially higher Lexical Diversity (MTLD: M = 190.83, SE = 7.82) than both human (M = 97.22, SE = 7.80; *p* < 0.001) and AI-assisted texts (M = 99.10, SE = 7.70; *p* < 0.001). Similarly, LLM texts were significantly more difficult (M = 1035.39, SE = 31.38) than human texts (M = 525.23, SE = 55.81; *p* < 0.001), though not significantly different from AI-assisted texts (M = 659.45, SE = 145.96; *p* = 0.011). Additionally, LLM texts showed lower syntactic efficiency (ADD: M = 3.15, SE = 0.05) compared to human (M = 3.45, SE = 0.09; *p* = 0.029) and AI-assisted (M = 3.47, SE = 0.11; *p* = 0.019) outputs. No significant differences emerged for Paragraph Similarity in this style after correction.

Plain Profile (low originality, mid-effectiveness): Despite limited human representation (n = 1), pairwise comparisons in the Plain style revealed that LLM texts used significantly fewer words per sentence (M = 22.55, SE = 0.40) than human texts (M = 36.00; *p* < 0.001) and AI-assisted texts (M = 24.14, SE = 3.40; *p* = 0.001). LLM texts also displayed lower syntactic efficiency (ADD: M = 3.23, SE = 0.04) relative to human texts (M = 4.24; *p* < 0.001). Furthermore, LLM texts were significantly more difficult (M = 1012.44, SE = 35.37) than AI-assisted texts (M = 687.48, SE = 141.45; *p* = 0.021).

Safe and Ideal Profile: In the Safe style, only Paragraph Similarity showed a robust interaction: LLM texts exhibited high internal paragraph coherence (M = 0.405, SE = 0.008), whereas both human and AI-assisted texts scored zero (*p* < 0.001 for both comparisons). In the Ideal style, no pairwise differences survived correction, though LLM texts showed numerically higher Paragraph Similarity (M = 0.415) than human texts (M = 0.000; *p* < 0.001).

Collectively, these findings indicate that LLM-generated texts diverged most markedly from human-authored texts when targeting moderate or plain creative strategies, characterized by tendencies toward higher lexical diversity, greater text difficulty, shorter sentences, and reduced syntactic fluency. In contrast, AI-assisted writing closely resembled human output across multiple features, particularly in the Moderate style.

## 5. Discussion

Our findings provide an empirical framework for resolving several key debates in the burgeoning field of comparative creativity. This discussion interprets these findings, situates them within the existing scholarly discourse, and elucidates their theoretical implications.

### 5.1. Task-Dependent Asymmetry of Originality: Embodied Cognition vs. Probabilistic Generation

The first principal finding of this study is that the human advantage in originality is not absolute but is context-dependent, surfacing primarily in high-demand tasks involving cognitively mature writers. The Human-Only group significantly outperformed the LLM-Only group (using state-of-the-art models) only in the high-demand creative task (CT), a speculative fiction task requiring complex conceptual blending and world-building. In the three propositional tasks (P1–P3), which rewarded argumentation and expository narrative, no significant difference was found.

This result provides powerful empirical validation for the theoretical dichotomy of human versus artificial creativity (Runco, 2024; Sternberg, 2024). Scholars have consistently argued that human creativity is “experience-driven” and “emotion-infused”, rooted in personal memory, embodied experience, subjective intent, and rich cultural context (Giannuzzo, 2023; Moura, 2023). This “subjective creativity” allows humans to make novel, cross-domain associations—for instance, linking “stone” to “symphony” or “quantum” to “nostalgia”—that are not based on statistical co-occurrence but on abstract, metaphorical, or emotional resonance.

In contrast, LLM creativity is described as “fact-driven” and “rule-constrained”, excelling at “objective creativity” (Boden, 2009). The propositional tasks, rewarding logical rigor and the novel combination of facts rather than subjective insight, fall squarely within this domain of objective, high-probability semantic generation. The LLM’s ability to synthesize vast patterns from its training data allowed it to match human performance in this context. This finding also resolves a tension in the literature regarding divergent thinking; while some studies suggest essay writing requires divergent thinking (which humans excel at) (Koivisto & Grassini, 2023), our data suggest that propositional essay writing may rely more on a form of high-level convergent synthesis that LLMs have mastered.

The observed human superiority in the Creative Task (CT) compared to the propositional tasks (P1–P3) serves as more than just a statistical boundary. We argue that this gap exists because LLMs, by their probabilistic nature, lack the “creative urge”—the internal motivation to disrupt conventions for a specific expressive purpose. While LLMs successfully simulate the “surface” of originality in constrained tasks, humans alone demonstrate the capacity for “radical originality” which stems from a history of lived experience and emotional tension. Our personal remark is that true creativity may remain an exclusively human province so long as AI lacks “intentionality”—the conscious “aboutness” of a narrative that guides every word choice toward a coherent, subjective vision.

### 5.2. The Universal Dominance of Effectiveness: The Perfection of "Safe" Writing

In stark contrast to the nuanced findings on originality, our study reveals an overwhelming and universal LLM advantage in effectiveness across all task types. LLM-generated texts were consistently rated as more coherent, grammatically flawless, and rhetorically compelling (Vinchon et al., 2024). This challenges early assumptions that AI struggles with “appropriateness” or “convergent thinking”; rather, our results—underpinned by enormous Cohen’s d values exceeding 1.0—suggest that modern LLMs exert a governing influence over the execution quality of writing. They have evolved beyond simple “optimized engines of appropriateness” to become dominant systems for structural rigor and formal effectiveness.

This dominance is mechanistically explained by the LLM’s training objective: perplexity minimization. By consistently predicting the most probable next token, LLMs naturally gravitate towards a “Safe Style” (low originality, high effectiveness) consisting of AI texts. This represents a form of “over-smoothing”, where the model prioritizes structural rigor and readability, effectively eliminating the “noise” of human error but also filtering out the “spikes” of radical novelty (O’Sullivan, 2025).

Conversely, the domination of the “Plain Style” (low originality, mid-effectiveness) by human authors highlights a “mediocrity trap”. This suggests that average human writers (represented here by 8th graders) often struggle with the executive control and structural planning that LLMs execute effortlessly. This reinforces the complementary potential of human–AI interaction: humans provide the “spark” of originality, while AI provides the “scaffold” of effectiveness.

However, this dominance brings a hidden cost—the “mediocrity trap”. Our analysis suggests that as AI raises the performance floor, it simultaneously exerts a powerful “gravitational pull” toward the average. Human writers, when faced with effortlessly perfect machine output, may succumb to “cognitive laziness”, prioritizing ease of generation over the difficult labor of finding a unique voice. We believe this represents a critical evolutionary crossroad for human authorship: we must learn to treat AI as a foundation to be transcended, not a ceiling to be reached.

### 5.3. Antithetical Linguistic Pathways: Cognitive Investment vs. Structural Optimization

Beyond output-based comparisons, this study makes a novel contribution by unpacking the “Black Box” of linguistic mechanisms. Our HMR analysis confirms that humans and LLMs utilize distinct and opposing linguistic pathways to achieve creativity.

For human authors, originality is predicted by linguistic markers of subjective cognitive investment.

Lexical Diversity (MTLD): In the propositional tasks, higher diversity predicted higher originality for humans, reflecting the cognitive effort of exploring a broader lexical space.

First-Person Subjectivity: In the creative task, the use of first-person singular pronouns was a powerful predictor of originality. This serves as a quantifiable linguistic marker for “authentic self-expression” and “agency” (Al Hosni, 2025), confirming that human creativity is inextricably linked to the assertion of a subjective voice (Giannuzzo, 2023).

For LLMs, effectiveness is driven by structural optimization.

Intra-Paragraph Similarity: The strong positive prediction of effectiveness by semantic similarity confirms that LLM competence relies on maximizing internal thematic consistency (O’Sullivan, 2025).

Complexity Penalty: Crucially, features that signaled human effort (e.g., high difficulty, high MTLD) had a negative or null effect on LLM performance. This implies that the LLM’s “Safe Style” is already at an effectiveness ceiling; forcing it toward human-like linguistic complexity breaks its optimized coherence, resulting in “stylistic noise” rather than depth.

### 5.4. The "Collaboration Trap": Semantic Collapse and Anchoring Effects

Our analysis of the LLM-Assisted (A) group in Phase 2 provided a nuanced and critical perspective on human–AI collaboration. This experiment, which constrained the human–AI partnership by using the suboptimal GPT-2 model (while the LLM-Only group used GPT-5), revealed a significant “collaboration trap”. Collaboration with a suboptimal LLM may inadvertently suppress human creative originality. This finding highlights the risks of “negative transfer” when humans partner with lower-performing systems, though it remains to be seen if this trap persists when collaborating with frontier models that possess higher structural and semantic sophistication.

The collaboration did not expand the creative territory; rather, it led to a regression toward the mean. While collaboration may boost individual productivity, our findings align with recent meta-analyses suggesting it reduces collective diversity (Doshi & Hauser, 2024). AI’s tendency to converge on “high-probability” regions of the training data (e.g., common metaphors) limits the variance of the final output, creating a “worst-of-both-worlds” scenario where humans inherit AI’s lack of genuine novelty without fully capitalizing on its structural perfection.

Despite failing to improve effectiveness, the A-group’s linguistic profile did change. Their texts adopted higher ParaSim, Difficulty, and Classical Words, shifting stylistically towards the LLM-Only profile (higher Difficulty and ParaSim) without the corresponding performance benefit. This provides direct empirical evidence for the “anchoring effect” and “semantic space collapse” that the literature warns about (Doshi & Hauser, 2024). Writers expended effort aligning with the tool’s suggestions, resulting in a text that was more “difficult” but not more “effective.” Our study shows this anchoring in a real-world writing task, where writers were anchored to a suboptimal AI’s style, resulting in a “worst-of-both-worlds” hybrid. 

However, the collaboration was not a total surrender of agency. Even while being structurally anchored, the A-group retained a high usage of First-Person Singular pronouns, matching the Human-Only group and significantly exceeding the LLM-Only group. This is a crucial finding that supports the “Preservation of Writer Values” hypothesis (Al Hosni, 2025). Our data suggest that even when collaborating with an algorithmic partner, human writers actively “set boundaries” to maintain authentic self-expression and subjective voice. They refused to cede the “I” in their narrative, even while adopting the machine’s structural complexity. This negotiation—sacrificing structural autonomy while fiercely defending subjective identity—emerges as a central, defining tension in the future of human–AI co-creativity.

The “collaboration trap” observed with GPT-2 illustrates the danger of the “anchoring effect” in human–AI partnerships. It is our personal observation that the most insidious risk of AI assistance is not the generation of “wrong” ideas, but the “narrowing of the imaginative horizon”. By presenting high-probability suggestions, AI subtly pre-empts the human search for low-probability, “surprising” associations. To avoid this, future collaborative pedagogies must focus on “critical prompting”—teaching writers to intentionally push AI toward its statistical edges rather than following its lead toward the safe center.

### 5.5. Limitations and Future Directions

The findings of this study should be interpreted within the context of its specific methodological boundaries. A limitation of this study is that task category and participant cohort were not fully orthogonalized across phases. Phase 1 involved 8th-grade students completing propositional writing tasks, whereas Phase 2 involved undergraduate students completing a high-demand creative writing task. As such, task category in this design reflects a bundled context incorporating both task demands and developmental appropriateness. Importantly, the primary analyses focused on within-task comparisons between human and LLM-generated texts, and interpretations regarding task dependency are therefore contextual rather than strictly causal. Future research using age-matched or longitudinal designs is needed to disentangle task effects from developmental factors more precisely. Second, the LLM-Only group used GPT-5, while the LLM-Assisted group used GPT-2. This discrepancy was not an oversight but rather an analytical design reflecting the technical and accessibility constraints present at the time of data collection. Consequently, the LLM-Only group functioned as the “upper bound benchmark” in this study, while the LLM-Assisted group tested human collaborative strategies under realistic technological constraints. The findings should be interpreted strictly within the context of collaboration with a suboptimal LLM, rather than being generalized to interactions with more advanced frontier models like GPT-5. Future research should build upon this by employing unified LLMs across all AI-involved conditions to evaluate the full potential of an optimal human–AI collaborative paradigm. Lastly, the localized nature of the human sample (recruited from specific educational tiers in China) is acknowledged as a contextual boundary. However, this does not diminish the significance of the results, as the study focused on the mechanistic divergence between human cognition and LLM probability, which is less likely to be influenced by specific regional factors than by general linguistic and cognitive structures. To enhance generalizability, future research should replicate this framework across diverse cultural and linguistic backgrounds.

## 6. Conclusions

Grounded in the standard definition of creativity, this study established a dual-mechanism framework for comparative writing creativity. This approach fundamentally shifts the scholarly discourse from the normative evaluation of “Who is better?” to the mechanistic investigation of “How do they differ?” Our multi-stage analysis provides robust empirical evidence that human and artificial creativity are not isomorphic; instead, they operate through antithetical processes that yield convergent surface results.

The primary findings of this research are summarized as follows. (1) Task-Dependent Asymmetry: Human superiority in originality is not an absolute trait but is strictly contingent upon task complexity and participant maturity, surfacing only in high-demand creative contexts. (2) Execution Dominance: LLMs do not merely assist in writing; they effectively govern the domain of formal effectiveness. The sheer magnitude of the effect sizes observed confirms that LLMs provide a level of structural coherence that “raises the floor” of writing performance to a near-flawless standard. (3) Divergent Pathways: Human originality is a product of “subjective reconstruction” driven by cognitive investment and personal voice, whereas LLM effectiveness is a result of “probabilistic optimization” focused on internal thematic consistency.

In the current panorama of AI studies, this study holds significant theoretical and practical weight. Mirroring our initial inquiry into the boundaries of “uniquely human” creativity, we conclude that human agency resides not in the mechanics of fluency, but in the courage of subjective expression. By identifying the “collaboration trap” and the risk of “semantic collapse”, this study cautions against a simplistic, additive view of human–AI co-creativity. We have demonstrated that collaboration with suboptimal LLMs can inadvertently suppress originality through cognitive anchoring, highlighting an urgent need to treat AI as a structural scaffold rather than an inspirational generator.

These findings suggest that human–LLM asymmetry in creativity is deeply embedded within specific educational and developmental contexts. Future research should employ age-matched designs to further isolate the unique impact of task type from cognitive maturity. Ultimately, this research constitutes an urgent mandate for educational reform: as machines master formal effectiveness, the pedagogical focus must pivot toward cultivating “high-order originality”—the conceptual blending and critical intent that remain the unique province of the human spirit.

## Figures and Tables

**Table 1:** Final cleaned sample size by writing task and author type.

Task Phase	Task Type	Author Type	Initial Sample	Final Cleaned Sample (N)
Phase 1	Propositional	Human-Only (H)	200	189
		Human-Only (H)	200	190
		Human-Only (H)	200	188
		LLM-Only (L)	600	600
Phase 2	Creative Fiction	Human-Only (H)	25	25
		LLM-Assisted (A)	25	25
		LLM-Only (L)	79	79
Total Corpus Size			1329	1296

Note: The LLM-Only group for the Creative Task (CT) had an initial sample of 79, which were all retained in the final cleaned sample. The LLM-Only texts for P1, P2, and P3 had 200 clean texts each, totaling 600 texts.

**Table 2:** Linguistic features used to predict writing creativity.

Domain	Feature	Operational Definition	Theoretical Rationale for Creativity
Lexical	MTLD (Measure of Textual Lexical Diversity)	The mean number of words needed before a lexical Type–Token Ratio (TTR) value of 0.72 is reached; averaged over consecutive samples.	High diversity can indicate rich vocabulary but may also lead to reduced thematic focus, impacting perceived originality and effectiveness.
	Text Difficulty	An index of text difficulty calculated based on character frequency and strokes.	Text difficulty is a proxy for processing fluency; moderate difficulty may be perceived as more thoughtful and effective.
	Count of Classical Chinese Words	The absolute count of words identified as belonging to classical Chinese lexicons.	Use of classical language signals erudition and stylistic flair, potentially enhancing originality.
Semantic	Perceptual Processes	The proportion of words related to seeing, hearing, or feeling (e.g., “see”, “listen”), based on the LIWC2015 dictionary.	Perceptual language creates vivid mental imagery, which can enhance both the vividness (effectiveness) and novelty (originality) of a text.
	Use of First-Person Singular	The proportion of first-person singular pronouns (e.g., “I”, “me”).	High usage may indicate subjectivity and personal narrative, which can affect perceived objectivity and originality depending on the task.
	Mean Concreteness	The average concreteness rating of all content words.	Concrete words are easier to process and evoke mental imagery. A balance of concrete and abstract language is often key to effective and creative writing.
	Causal Language	The proportion of words indicating causal relationships (e.g., “because”, “effect”), based on LIWC2015.	Reflects logical reasoning and structure, which is crucial for the effectiveness of argumentative or expository texts.
	Family-Related Words	The proportion of words referring to family members (e.g., “mother”, “brother”), based on LIWC2015.	May indicate the use of personal or social themes, influencing the perceived relatability and style of the text.
Syntactic	Mean Parse Tree Height	The average maximum path length from the root to any leaf node in a constituency parse tree for each sentence.	A proxy for syntactic complexity; higher trees suggest more complex sentence structures, often associated with higher cognitive ability.
	Average Dependency Distance	The average linear distance (in words) between a headword and its dependent elements in a sentence.	A well-validated metric of syntactic complexity; greater distances suggest more complex phrasing and embedded clauses.
	Words Per Sentence	The total number of words divided by the total number of sentences.	Measures sentence length; longer sentences can convey more complex ideas but may reduce readability and effectiveness.
	Conjunctions	The proportion of conjunctions (e.g., “and”, “but”) used in the text, based on LIWC2015.	Reflects the complexity of logical connections between ideas.
Discourse	Inter-Sentence Similarity	The mean cosine similarity between consecutive sentence pairs, calculated using Sentence-BERT (SBERT) embeddings.	Measures local cohesion. Very high similarity may indicate repetition (low originality), while very low similarity may signal incoherence (low effectiveness).
	Intra-Paragraph Sentence Similarity	The mean cosine similarity between all sentence pairs within a paragraph, calculated using SBERT embeddings.	Measures thematic cohesion at the paragraph level, a key component of overall text effectiveness.

Note. LIWC2015 = Linguistic Inquiry and Word Count 2015 dictionary (Pennebaker et al., 2015); SBERT = Sentence-BERT, a sentence embedding model; TTR = Type–Token Ratio. Operational definitions are standardized per cited methodologies. The theoretical rationale links each feature to established constructs in writing research, cognitive psychology, and computational linguistics.

**Table 3:** Summary of two-way ANOVA for originality and effectiveness scores.

Dependent Variable	Independent Variable	Sum of Squares	df	F-Statistic	*p*-Value	Partial η^2^
Originality	Author Type (A)	0.023	1	0.059	0.808	<0.001
(N = 1256)	Task Category (T)	0.020	1	0.050	0.823	<0.001
	A × T Interaction	3.693	1	9.399	0.002	0.007
	Residual	492.011	1252			
Effectiveness	Author Type (A)	107.061	1	328.068	<0.001	0.208
(N = 1256)	Task Category (T)	16.329	1	50.036	<0.001	0.038
	A × T Interaction	0.064	1	0.196	0.658	<0.001
	Residual	408.574	1252			

**Table 4:** Summary of independent sample *t*-tests for pairwise comparisons (Human-Only vs. LLM-Only).

Task	Dimension	*t*	df	*p*-Value	Cohen’s d	Key Finding
Creative Task (CT)	Originality	3.764	102	<0.001	0.74	H > L
	Effectiveness	−4.280	102	<0.001	0.84	L > H
Propositional P1	Originality	−1.407	387	0.160	−0.14	Not significant
	Effectiveness	−9.459	387	<0.001	−0.96	L > H (Large Effect)
Propositional P2	Originality	0.863	388	0.389	0.09	Not significant
	Effectiveness	−9.502	388	<0.001	−0.97	L > H (Large Effect)
Propositional P3	Originality	−0.463	386	0.643	−0.05	Not significant
	Effectiveness	−11.622	386	<0.001	−1.18	L > H (Very Large Effect)

**Table 5:** Mean scores and size for the four creativity profiles (N = 1265).

Cluster Index	Cluster Name (Profile)	Mean Originality (SD)	Mean Effectiveness (SD)	Sample Size (N)	Percentage (%)
0	Ideal Style (High O, High E)	3.75 (0.35)	3.87 (0.46)	286	22.61
1	Plain Style (Low O, Mid-E)	2.26 (0.27)	3.28 (0.34)	332	26.24
2	Safe Style (Low O, High E)	2.80 (0.30)	3.58 (0.40)	448	35.41
3	Moderate Style (Mid-O, Low E)	3.30 (0.31)	2.59 (0.35)	199	15.73

Note: Originality and effectiveness scores range from 1 to 5. The total sample size for the clustering results presented here is N = 1265 due to data filtering.

**Table 6:** Author type distribution within each creativity profile.

Author Type	Ideal (High O, High E)	Moderate (Mid-O, Low E)	Safe (Low O, High E)	Plain (Low O, Mid-E)
Human (Student)	49.82%	49.85%	26.62%	84.92%
LLM (AI-only)	50.18%	50.15%	73.38%	15.08%

**Table 7:** Summary of the results for all models where the interaction term was statistically significant.

Task Category	Dependent Variable	Linguistic Feature	ΔR^2^	Β Interaction	*p*-Value
Propositional	Effectiveness (E)	Difficulty	0.016	−0.001	<0.001
	Effectiveness (E)	Classical Words	0.008	−0.005	<0.001
	Effectiveness (E)	Percept	0.0072	−9.321	0.001
	Effectiveness (E)	MTLD	0.0059	−0.002	0.003
	Originality (O)	MTLD	0.0328	−0.005	<0.001
	Originality (O)	Difficulty	0.0056	−0.001	0.009
	Originality (O)	Family Words	0.0055	9.738	0.012
Creative	Effectiveness (E)	Difficulty	0.0594	−0.002	0.007
	Effectiveness (E)	Paragraph Similarity	0.0000	2.087	0.008
	Originality (O)	First-Person Singular	0.0469	15.40	0.019
	Originality (O)	Difficulty	0.0366	−0.001	0.04

**Table 8:** Sample distribution across author type and creative style in the CT.

Author Type	Ideal (C0) (High O, High E)	Plain (C1) (Low O, Mid-E)	Safe (C2) (Low O, High E)	Moderate (C3) (Mid-O, Low E)	Total
Human (HO)	7	1	1	16	25
AI-Assisted (HAC)	5	3	1	16	25
LLM-Only (LO)	10	26	25	18	79
Total	22	30	27	50	129

**Table 9:** Creative task mean score comparison and ANOVA.

DV	Mean (H)	Mean (A)	Mean (L)	F	*p*-Value	Tukey HSD (Adj.)
Originality	3.20	3.10	2.93	1.55	0.220	No Significant Differences
Effectiveness	2.87	2.82	3.82	32.73	<0.001	L ≫ H; L ≫ A

**Table 10:** Significant linguistic feature means and ANOVA results in creative task.

Feature	Mean (H)	Mean (A)	Mean (L)	F	*p*-Value
ParaSim (Intra-Paragraph Similarity)	0.000	0.318	0.417	103.70	<0.001
First-Person Singular	0.032	0.044	0.013	17.64	<0.001
Difficulty	589.997	749.099	877.545	5.27	0.007
Concreteness	2.476	2.453	2.560	9.14	<0.001
Classical Words	90.240	114.320	126.933	3.27	0.044
Conjunctions	0.046	0.057	0.047	3.85	0.025

**Table 11:** Two-way ANOVA results for language features with significant author type × style interaction.

Feature	F(6, 114)	*p*	Partial η^2^
Paragraph Similarity (Adj.)	3.40	0.004	0.148
Lexical Diversity (MTLD)	2.64	0.020	0.119
Difficulty	2.46	0.028	0.112
Words Per Sentence	2.40	0.032	0.110

## Data Availability

The datasets presented in this article are not readily available because they belong to an ongoing research project. Requests to access the datasets should be directed to the author at lipingyoung@163.com.

## Appendix A. Writing Task Prompts

Task 1: Propositional Writing Tasks

Instructions: Write an essay of over 400 characters, focusing on a clear central theme and genuine emotion. Genre is unrestricted.

- Topic 1: Companionship is the Best Gift;
- Topic 2: If I Could Do It Again;
- Topic 3 (Title Completion): I Forgot ________.

Task 2: College Creative Writing (Design Fiction Workshop)

This task was designed for second-year science and engineering students participating in a Design Fiction workshop.

- Workshop Theme:

To critically reflect on the relationship between TECHNOLOGY and HUMAN.

B. Core Task: Design Fiction.

1. Possible Forms: An allegorical story, a topic for discussion, or a question full of contradiction/doubt.
2. Creation Basis: Based on speculation and imagination, construct a future scenario set in the year 2040.
3. Two Proposed Contextual Assumptions for 2040 (Choose One):
  - The Era of AI Involution (Hyper-competition): By 2040, AI and automation have replaced some human jobs, boosting productivity but intensifying “Involution” (hyper-competition). Competition extends between humans, between humans and AI, and between AIs (e.g., the “Involution King” competes with AI, and some automation is developed solely for the sake of hyper-competition).
  - Data Workers: By 2040, AI and automation have replaced some human jobs, work types have diversified, and “living is working” has become the norm. Humans generate data through daily activities (including sleep) to earn income, with different behaviors priced differently.

C. Four-Step Creation Process

Step 1: Select a Background and Create Scenes

- Choose one 2040 background assumption and generate 4–5 imaginative scenes, using a fixed sentence structure for each:
- “In 2040, a ___________ (what kind of) scenario occurred. I ___________ (what situation/difficulty/challenge/event did I encounter). I hope ___________ (what change I want to make/what goal I want to achieve).”

Step 2: Expand on Action

- Based on the “future snippet,” supplement the action: “…To achieve this goal, I then did ______________” (multiple imaginative and contextually relevant actions may be developed).

Step 3: Analyze Impact and Results

- Select the most preferred snippet and elaborate on possible results/impacts (can involve personal/societal levels, be positive/negative, be expected/unexpected, or be active/frustrating).

Step 4: Form a Complete Story (Approx. 400 Chinese Characters)

- Elaborate on the snippet to form a complete Design Fiction, which must include the following:
  - The social context of 2040;
  - Events that occurred in 2040 (characters, actions, new products/services/policies);
- Reflection on the critical issues (impact of technology on work, the meaning of work for humans, the boundaries of work, etc.; reflection focus: AI’s impact on humans, ideal AI form, AI’s impact on future work, essential qualities of meaningful work, or division of labor between AI and humans).

## Appendix B. Creativity Rating Instrument and Guidelines

The ratings were conducted using a 5-point Likert scale, where 5 indicated “Strongly Agree” (Very High Creativity) and 1 indicated “Strongly Disagree” (Very Low Creativity).

1. Rating Dimensions and Descriptors

**Table A1:** Rubric of writing creativity.

Dimension	Scale	5 (Strongly Agree/Very High) Descriptors
Originality	5-1	The plot or basic idea is original, i.e., it is very different from ordinary essays and other familiar stories (from books, movies, TV, myths, etc.). The text contains something special or original. The fictional technology, objects, and events demonstrate strong imagination and creativity. It contains unexpected elements (e.g., plot development, ideas, design, etc.).
Effectiveness	5-1	The premise of Design Fiction is that the fictional technology, objects, and events are not magical but technically plausible. The overall narrative of the fictional creation feels logical and self-consistent to the reader. The reader can perceive a link between the fictional creation and certain trends in the real world.

2. Detailed Explanations and Guidelines

- Novelty Aspects (More Detailed Definitions)

Novelty can manifest in various ways; the following are some examples:

- Unusual Character or Background: The story’s participants or background are highly unusual (e.g., in the topic “Companionship is the Best Gift,” a “book” is a more novel subject than “parents”). Characters or settings are very unusual (e.g., animals or aliens are main characters; the story is set on another planet). The author makes an unusual or unexpected choice in interpreting the writing prompt (e.g., literally interpreting the concept of power, or interpreting it from a drastically different angle than typical explanations).
- Unusual Plot or Development: An unusual or unexpected development of the story or plot (e.g., describing a “father who left home early and did not accompany the grandmother”). The author makes unusual or unexpected choices in the plot’s direction (e.g., the story begins like a corny teen romance but has a surprisingly dark ending; the story starts very realistically but ends unexpectedly mysteriously; the story begins very dark but unexpectedly becomes funny).
- Uncommon Thoughts or Emotions: The author expresses uncommon but appropriate thoughts, emotions, or judgments (e.g., a “left-behind” child’s longing for a complete family).
- Rare Language Style: The author chooses very rare words, phrases, language, or rhetorical styles (e.g., extensive parallelism, personification, or archaic Chinese language).

B. Concreteness Aspects (Detailed Definitions)

- Abstract: Refers to concepts that cannot be experienced through the senses (e.g., love and despair).
- Generalization: Use of overly vague and un-visualized terminology (e.g., “everything” or “her life”) without further explanation.

C. Exclusion Criteria

- Off-topic essays are marked “Off-topic,” and blank papers are marked “Blank.” Neither receives a score.
- No ideological or moral judgments are to be made.
